# suPAR as a prognostic biomarker in sepsis

**DOI:** 10.1186/1741-7015-10-2

**Published:** 2012-01-05

**Authors:** Katia Donadello, Sabino Scolletta, Cecilia Covajes, Jean-Louis Vincent

**Affiliations:** 1 Department of Intensive Care, Erasme University Hospital, Université Libre de Bruxelles, 808, route de Lennik 1070, Brussels, Belgium

**Keywords:** infection, sepsis, biomarker, disease severity, case fatality, outcome, soluble urokinase-type plasminogen activator receptor, suPAR

## Abstract

Sepsis is the clinical syndrome derived from the host response to an infection and severe sepsis is the leading cause of death in critically ill patients. Several biomarkers have been tested for use in diagnosis and prognostication in patients with sepsis. Soluble urokinase-type plasminogen activator receptor (suPAR) levels are increased in various infectious diseases, in the blood and also in other tissues. However, the diagnostic value of suPAR in sepsis has not been well defined, especially compared to other more established biomarkers, such as C-reactive protein (CRP) and procalcitonin (PCT). On the other hand, suPAR levels have been shown to predict outcome in various kinds of bacteremia and recent data suggest they may have predictive value, similar to that of severity scores, in critically ill patients. This narrative review provides a descriptive overview of the clinical value of this biomarker in the diagnosis, prognosis and therapeutic guidance of sepsis.

## Introduction

Sepsis is defined as the clinical syndrome resulting from the presence of both infection and a systemic inflammatory response [1]. Sepsis involves the activation of inflammatory and anti-inflammatory mediators, cellular and humoral reactions, and micro- and macro-circulatory alterations. Despite improvements in the management of critically ill patients with serious infections, sepsis is still the leading cause of death in critically ill patients [2]. Early diagnosis of sepsis is vital because rapid, appropriate therapy is associated with improved outcomes [3]. There is, therefore, a need for better techniques to facilitate the diagnosis of sepsis and to monitor its course. Various biomarkers, biological molecules that are characteristic of normal or pathogenic processes and can be easily and objectively measured, have been proposed as being of potential use for sepsis diagnosis, therapeutic guidance, and/or prognostication [4,5], although their exact role remains undefined [3]. The two biomarkers that have been most widely studied and used in patients with sepsis are C-reactive protein (CRP) and procalcitonin (PCT). Levels of both these biomarkers have been demonstrated to be raised in patients with sepsis making them useful diagnostic indicators [6,7]. Importantly, because they lack specificity for sepsis and levels may be raised in other inflammatory diseases, these biomarkers are more useful for ruling out sepsis than for ruling it in, that is, a completely normal value makes a diagnosis of sepsis very unlikely. PCT, in particular, has also been used for therapeutic guidance in patients with various types of infection [7].

Recently, the soluble form of the urokinase-type plasminogen activator receptor (suPAR) has attracted scientific interest because it seems to discriminate better than some other biomarkers among patients with different severities of illness [8]. In this narrative review, we discuss the available literature on suPAR in sepsis and provide a descriptive overview of the clinical value of this biomarker in the diagnosis, prognosis and therapeutic guidance of sepsis.

### Structure and history of suPAR

**Figure 1:**
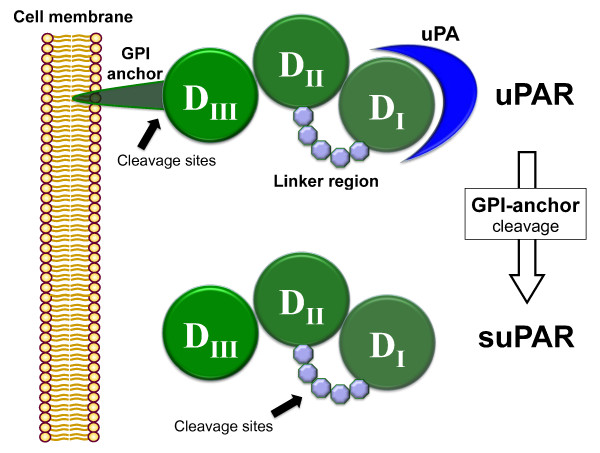
**Schematic of the structure of uPAR, the mechanism of cleavage and the formation of suPAR**. DI, DII, DIII represent the three homologous domains of suPAR.

The urokinase-type plasminogen activator (uPA) system consists of a protease, a receptor (uPAR) and inhibitors. In 1990, uPAR was cloned [9] and, in 1991, Ploug *et al*. identified its soluble form (suPAR) [10]. uPAR is expressed on various cell types including neutrophils, lymphocytes, monocytes/macrophages, endothelial and tumor cells. After cleavage from the cell surface, suPAR can be found in the blood and other organic fluids in all individuals, existing in three forms (I-III, II-III and I) that have different properties related to their structural differences (Figure 1) [11]. suPAR takes part in various immunological functions, including cell adhesion, migration, chemotaxis, proteolysis, immune activation, tissue remodeling, invasion and signal transduction [12]. Serum concentrations are stable throughout the day with limited circadian changes and are not influenced by fasting. Cerebrospinal fluid (CSF), urine and serum (after centrifugation of whole blood) levels can be measured with a monoclonal antibody double sandwich enzyme-linked immunosorbent assay (ELISA) using commercial kits (for example, R &D Systems, Minneapolis, MN; suPARnostic™, Virogates, Copenhagen, Denmark). In healthy adults, the median value of suPAR has been cited as 1.5 ng/ml (range: 1.2 to 1.9 ng/ml, N = 44) [13], or 2.6 ng/ml (range: 1.5 to 4.0 ng/ml, N = 31) [14].

### suPAR as a diagnostic marker of sepsis

As early as 1995, elevated plasma suPAR levels were reported in a small group of septic intensive care unit (ICU) patients [15]. During endotoxemia, suPAR expression is increased on peripheral blood mononuclear cells [16] as on monocytes and granulocytes [17,18]. However, although suPAR serum concentrations were increased after administration of high-dose endotoxin [16], low-dose endotoxin did not significantly increase plasma suPAR levels *in vivo *[16]. On the other hand, PCT and CRP are strongly induced by endotoxin [19-21], which may explain their enhanced usefulness for the diagnosis of bacterial infection.

In 100 patients with Crimean-Congo hemorrhagic fever (CCHF) [22], serum suPAR levels were significantly higher in patients with infection than in healthy controls; the optimum diagnostic cut-off value was 3.06 ng/ml, with an area under the receiver operating characteristic curve (AUROC) of 0.94. In a cohort of 156 patients with suspected sepsis [23], 96 of whom had bacterial infection, AUROCs for diagnosis of bacterial sepsis were 0.72 for PCT, 0.81 for CRP and only 0.50 for suPAR levels, suggesting that suPAR was of less value for diagnosis than these other biomarkers.

suPAR levels may be measured in other milieu than blood. In 183 patients clinically suspected of having meningitis, suPAR levels were significantly higher in the CSF of patients with proven central nervous system (CNS) infection than in those without [24]. There were no differences in CSF suPAR levels between patients with meningitis and those with encephalitis but levels were significantly higher in patients with purulent (especially in pneumococcal infection) than in those with lymphocytic meningitis. A cut-off value of 1.50 mcg/l distinguished purulent from viral meningitis. Similar findings were reported in a smaller study of just 12 patients with bacterial meningitis [25]. Nevertheless, because of the relatively low diagnostic sensitivity and specificity, the routine use of CSF suPAR measurement in CNS disease cannot be recommended at the present time.

A study by Koch *et al*. [26] is currently the only published report evaluating the diagnostic and prognostic impact of suPAR in a large cohort of critically ill patients (n = 273). Critically ill patients had higher serum suPAR concentrations at admission than healthy controls. The AUROC for prediction of sepsis was 0.62, compared to 0.86 for CRP and 0.78 for PCT. suPAR concentrations were closely related to other sepsis markers, including CRP, PCT, and tumor necrosis factor α levels. suPAR levels were also inversely related to renal function (as assessed by cystatin C, creatinine or urea levels), reflecting the renal clearance of suPAR; they were inversely related to albumin, and directly related to markers of cholestasis (for example, bilirubin, alkaline phosphatase). In an ongoing study in critically ill patients [27], we have found that a cut-off value of 5.5 ng/ml has a sensitivity of 75% and specificity of 72% for diagnosing sepsis. In this study, suPAR levels were correlated to CRP levels in the whole study population, but not in the group of patients with sepsis.

Table 1 summarizes the available data on the diagnostic value of suPAR in sepsis. Taking all these results into consideration, it appears that suPAR has poor accuracy in diagnosing sepsis compared to CRP and PCT, making suPAR of limited value as a diagnostic marker of sepsis.

**Table 1:** Studies evaluating the diagnostic value of soluble urokinase-type plasminogen activator receptor (suPAR) levels

First author, publication date [ref]	Type	Pathology	Patients	Period	Main results	Comments
Kofoed, 2007 [23]	Prospective	Suspected sepsis	156 adult, samples taken at ED admission	12 months	AUC bacterial sepsis: suPAR 0.5, PCT 0.72 CRP 0.81	
Yilmaz, 2010 [22]	Retrospective	CCHF	100 infected adult pts vs volunteers. Samples taken at hospital admission	2006-2009 38 months	Patients (6.2 ± 4.2 ng/ml) versus controls (2.3 ± 0.6 ng/ml), *P *< 0.0001. Cut-off 3.06 ng/ml AUC 0.94 (PPV 95%, specificity 92%)	No other infections studied
Østergaard, 2004 [24]	Prospective	CNS infection	183 adults, samples taken at admission	1988 to 2002	Higher CSF levels in infected patients and in patients with purulent meningitis versus those with lymphocytic meningitis (*P *< 0.001)	Low sensitivity and specificity (69% and 71%) with cut-off value of 1.50 mcg/l
Koch, 2011 [26]	Prospective	Critical illness medical ICU	273 adults, 197 septic patients, samples taken at ICU admission	Undefined	AUC sepsis suPAR 0.615 PCT 0.857 CRP 0.780	Correlation with renal and hepatic function
Donadello, 2011 [27]	Prospective	Critical illness medico-surgical ICU	152 adults, 55 septic patients. Samples taken at ICU admission	December 2010 to March 2011	AUC sepsis 0.75 (95% CI 0.66 to 0.83); correlation with CRP in global population (r = 0.48), not in septic patients (r = 0.18)	Preliminary data

AUC, area under the curve; CCHF. Crimean Congo Hemorrhagic Fever; CNS,central nervous system; CRP, C- reactive protein; CSF, cerebrospinal fluid; ED, emergency department; PCT; procalcitonin; PPV, positive predictive value.

### suPAR as a prognostic biomarker in sepsis

Biomarkers are relevant in clinical practice not only for their ability to diagnose a pathological condition, but also for predicting morbidity and outcome. Several studies have indicated that suPAR concentrations may reflect the severity of infection and have reported that they are associated with a worse outcome in a range of non-infectious and infectious diseases (Table 2). An association with mortality has been reported in patients with malaria [28], tuberculosis [29], and human immunodeficiency virus (HIV) infection [30-32]. In a study of 314 HIV-1 infected patients, the median serum suPAR value was 3.69 ng/ml [33]. Serum levels were higher in patients with lower CD4 counts, higher viral loads, and a higher incidence of AIDS-related death. There was a weak but significant negative correlation between suPAR levels and CD4 count, and a weak positive correlation between suPAR levels and viral load. The survival curves were significantly different for patients with low, medium and high suPAR levels, showing lower survival rates as suPAR levels increased. In a multivariate Cox regression model, suPAR levels were a stronger predictor of survival than CD4 count and viral load [33].

**Table 2:** Studies evaluating the prognostic value of soluble urokinase-type plasminogen activator receptor (suPAR) levels

First author	Type	Pathology	Patients	Period	Main results	Comments
Sidenius, 2000 [33]	Retrospective	HIV	314 adults, samples taken at enrollment	1991 to 1992	Range of suPAR levels 1.15 to 15.60 ng/ml. Low (< 3.28 ng/ml), medium (3.28-4.19 ng/ml) and high (> 4.19 ng/ml) suPAR levels related to increasing risk of AIDS-related death. Hazard ratio for death was 2.2 for medium suPAR levels (vs low) and 4.7 for high suPAR levels	Samples were not all obtained at enrollment
Eugen-Olsen, 2002 [29]	Retrospective	Mycobacterium tuberculosis	262 adults, samples taken at enrollment in a cohort based on suspicion of active tuberculosis 8 month-follow-up for 101 patients	1996 to1998	Elevated levels in active TB. 1.25 increase in mortality per ng increase in suPAR.	Not all patients were followed-up
Ostrowski, 2005 [30]	Prospective	HIV	59 healthy individuals + 99 HIV patients. Samples taken at study inclusion-median time from first positive HIV antibody test was 8 (5 to 9) years	2000 to 2001	Higher levels predicted increased mortality risk. suPAR(I-III) and (II-III) are independent predictors of mortality	Measurement of suPAR (I-III),(II-III) and (I) forms
Ostrowski, 2005 [28]	Prospective	Malaria	645 African children with clinical symptoms of malaria: 478 had malaria.14 healthy children as controls. Samples taken at hospital admission.	June to August of 2000 and 2001	Highest concentrations in non-survivors (11) or with complicated malaria. 1 ng/mL increase in suPAR concentration was associated with increased mortality (OR 1.42)	Low platelet count and hemoglobin level, high neutrophil count were independent predictors of high plasma concentration of suPAR
Lawn, 2007 [32]	Prospective	HIV	293 adults. Samples taken at enrollment for antiretroviral treatment	Sept 2002 to Feb 2005 5 month follow-up after enrollment	Significantly higher suPAR levels in non survivors. Log10 suPAR strongly associated with death	No discriminatory cut-off point to provide clinically useful information
Yilmaz, 2010 [22]	Retrospective	CCHF	100 adults, samples taken at hospital admission	2006 to 2009 38 months	Cut-off value of 10.6 ng/ml AUC 0.97	Only 5/100 deaths No comparison with other infections
Kofoed, 2008 [34]	Retrospective sample analysis	Suspected sepsis 64% bacterial infection	151 adults, samples taken at ED admission	12 months	Mortality: suPAR AUROC 0.80 (sensitivity 89%, specificity 63%, 95% CI 0.69-0.92). suPAR and age AUROC 0.92 (sensitivity 100%, specificity 78%, 95% CI 0.86-0.97)	PCT and CRP had no prognostic value
Ostergaard, 2004 [24]	Prospective	CNS infection	183 adults. Samples taken at admission	1988 to 2002	Positive correlation of CSF suPAR levels with prognosis; cut-off 3.1 mcg/l had OR for death of 11.9 (95% CI 1.4-106)	Multivariate analysis was not possible due to small number of deaths
Wittenhagen, 2004 [14]	Multicenter prospective study	*S. Pneumonia *bacteremia	141 adults. Samples taken at hospital admission	1999 to 2001; 21 months	Higher suPAR levels in patients compared to healthy volunteers (median 5.5, range 2.4 to 21.0 ng/ml). Levels > 10 ng/ml independent predictor of mortality (OR 13, specificity 95%, sensitivity 38%, NPV 88%, PPV 60%)	Logistic multivariate regression analysis
Huttunen, 2011 [8]	Prospective cohort study	Bacteremia	132 adults. Samples taken at day 1 after the first positive blood culture	June 1999 to Feb 2004	11 ng/ml AUROC 0.84 (95% CI 0.76 to 0.93, sensitivity 83%, specificity 76%). Higher levels associated with disease severity. OR for mortality16.1 (95%CI 4.3 to 59.9-logistic regression analysis)	Plasma samples were not taken at admission
Molkanen, 2011 [36]	Retrospective sample analysis	*S. aureus *bacteremia	59 adults. Samples taken on day 3, after positive blood culture		suPAR AUROC for mortality 0.754 (95% CI 0.615 to 0.894, *P *= 0.003) CRP AUROC 0.596. Cut-off 9.25 ng/ml	Plasma samples not taken at admission
Koch, 2011 [26]	Prospective	Critical illness medical ICU	273 adults, 197 septic. Samples taken at ICU admission	Undefined	Correlation of suPAR levels with APACHE II score (r = 0.345, *P *< 0.001), SOFA score (r = 0.337, *P *= 0.004), SAPS II score (r = 0.271, *P *= 0.004) and the need for VP and MV. Unadjusted OR for mortality 1.07 (95% CI 1.02 to 1.11) Cut-off value for mortality 8 ng/ml (day 1) to 13 ng/ml (day 3)	AUROC for ICU/overall survival larger (0.68/0.64) than CRP (0.52/0.53), PCT (0.55/0.55) and APACHE II (0.54/0.60), smaller than SAPS2 (0.81/0.74)
Donadello, 2011 [27]	Prospective	Critical illness, medico-surgical ICU	152 adults, 55 septic. Samples taken at ICU admission	December 2010 to March 2011	Cut-off value 6 ng/ml (sensitivity 63%, specificity 60%). AUROC for mortality 0.71 (95% CI 0.60 to 0.81) in overall population, in septic patients 0.68 (95% CI 0.47 to 0.88)	Preliminary data

AIDS: acquired immunodeficiency syndrome; APACHE II, Acute Physiology And Chronic Health Evaluation II; AUROC, area under the receiver operating characteristic curve; CCHF, Crimean Congo Hemorrhagic Fever; CNS, central nervous system; CRP, C-reactive protein; CSF, cerebrospinal fluid; ED, emergency department; MV, mechanical ventilation; NPV, negative predictive value; PCT, Procalcitonin; PPV, positive predictive value; OR, odds ratio; SAPS, Simplified Acute Physiology Score; SOFA, sequential organ failure assessment; TB, tuberculosis; VP, vasopressors.

In a small series of patients with CCHF, serum suPAR levels were related to renal and hepatic function and were of prognostic value [22]. No analysis for confounding factors was made by the authors of this study, but recently, using linear regression analysis, Koch *et al*. [26] showed that renal and liver function were independent predictors of elevated suPAR levels. CSF suPAR levels in patients with meningitis were positively correlated with age, CSF leukocyte and neutrophil count, CSF/blood-glucose ratio, altered Glasgow Coma Scale score, and need for assisted ventilation [24]. CSF suPAR levels were also higher in non-survivors compared to survivors.

Kofoed *et al*. [34] compared the prognostic value of suPAR to that of other biomarkers (soluble triggering receptor expressed on myeloid cells (sTREM-1) and macrophage migration inhibitory factor (MIF)) and of the Simplified Acute Physiology Score (SAPS) II and Sequential Organ Failure Assessment (SOFA). Of 151 patients with possible sepsis, 64% had a bacterial infection. suPAR levels (measured using the suPARnostic assay, cut-off value > 6.61 mcg/L) had a better prognostic value than PCT and CRP, equal to that of the admission SOFA score and almost as good as the SAPS II score; the combination of suPAR and age had a better prognostic value than the SAPS II score alone.

In a multicenter prospective study of 141 adult patients with *Streptococcus pneumoniae *bacteremia, Wittenhagen and colleagues [14] found that suPAR levels at admission were significantly increased compared to those of healthy controls. suPAR levels were higher in the 17% of patients who died from the infection than in those who survived. In a logistic multivariate regression analysis including clinical variables with a prognostic value (hypotension, renal failure, cerebral symptoms at admission, alcohol abuse), only suPAR levels above 10 ng/ml independently predicted mortality. The very high suPAR levels were similar to those found in patients with Gram-negative urosepsis [35] and in patients with bacterial meningitis [25].

Huttunen and colleagues [8] investigated suPAR levels as a predictor of disease severity and mortality in 132 patients with bacteremia caused by *Staphylococcus aureus*, *Streptococcus *(pneumonia and β-hemolytic) or *Escherichia coli*. The best mortality predictive cut-off level was 11 ng/ml. During the 30-day follow-up period, 18 patients died; 15 of them had suPAR levels above 11 ng/ml, compared to only three with levels below 11 ng/ml. Levels above this cut-off were also associated with disease severity (hypotension, need for vasopressors or mechanical ventilation, SOFA score ≥ 4). Logistic regression analysis gave an odds ratio for mortality of 16.1 (95% confidence interval [CI] 4.3 to 59.9), which remained significant after adjustment for potential confounders (for example, liver disease and renal failure). Interestingly, a simple suPAR measurement predicted mortality at least as well as the SOFA score. High suPAR levels were similarly demonstrated to predict mortality in a small cohort of 59 patients with *S. aureus *bacteremia [36]. Serum suPAR levels on day 3, after the first positive blood culture for *S. aureus*, were higher in the 19 patients who did not survive than in the 40 survivors and this difference persisted for 10 days. The best cut-off value was 9.25 ng/ml.

In the study by Koch *et al*. in critically ill patients [26], suPAR levels were strongly linked to disease severity scores, such as Acute Physiology and Chronic Health Evaluation II (APACHE II), SOFA, and SAPS II scores, and with the need for mechanical ventilation and vasopressor support. Moreover, low suPAR levels at ICU admission, and on days 3 and 7 were strong predictors of ICU survival (ICU mortality = 28%); after multivariate Cox regression analysis, suPAR levels retained a significant prognostic value. The best cut-off values for ICU survival were 8 ng/ml at day 1 and 13 ng/ml at day 3. The AUROC for ICU/overall survival was larger for suPAR than for CRP, PCT and the APACHE II score, but smaller than for the SAPS II score. In our study in a mixed ICU cohort of patients we found that a cut-off value of 6 ng/ml had 63% sensitivity and 60% specificity for predicting ICU mortality with an AUROC of 0.71, but this was less than that of the APACHE II and SOFA scores. The suPAR AUROC for ICU mortality in septic patients was 0.68 [27].

Importantly, when interpreting the role of suPAR as a prognostic marker from the results of these different studies, it is important to take into consideration the time of blood sampling for suPAR measurement, as a stratification biomarker that is robust during the first days of admission is probably more useful than one that provides a prediction later in the ICU course. In summary, high levels of suPAR have been widely demonstrated to correlate with morbidity and outcome, supporting its value as a prognostic biomarker in various cohorts of infected patients; moreover several studies have reported that values greater than 10 ng/ml may be predictive of death.

### suPAR for monitoring response to treatment

Another potentially important use for a sepsis biomarker is in monitoring response to treatment. Adequate antimicrobial therapy is an essential aspect of management in patients with sepsis but duration of antimicrobial therapy is poorly defined. Prolonged and unnecessary use of antibiotics is associated with increased costs, adverse effects, and development of antimicrobial resistance. Hence, being able to follow response to therapy and guide antimicrobial use could be of value, as has been suggested for other biomarkers [37]. In this context, suPAR levels were followed in HIV patients enrolled in an antiretroviral treatment (ART) program in South Africa [32]. Plasma suPAR levels were the strongest independent predictor of short-term mortality risk but the results did not permit determination of a discriminatory threshold that could be used to triage patients. In patients with extra-pulmonary mycobacterial infection, suPAR levels remained elevated for more than six months during adequate anti-mycobacterial therapy [38], probably reflecting prolonged inflammatory activity in these patients. Furthermore, in a large cohort of critically ill patients, suPAR levels remained elevated during the first week of ICU treatment [26]. In a cohort of young children suspected of having *Plasmodium falciparum *malaria, levels of suPAR were decreased significantly after seven days of effective treatment compared to admission levels [39].

The available data suggest that sequential suPAR levels may be of use in following the acute response to treatment in patients with sepsis. However, the results from these relatively small studies need to be further validated in larger, multicenter trials before this approach can be recommended. Moreover, the heterogeneous nature of the current studies prevents any meta-analytic technique to derive an optimal range of values for prognostication.

## Conclusions

The studies that have evaluated suPAR levels vary in the types of patient populations studied, the basal conditions of the patients, and the methods used to measure suPAR. The precise pathogenic involvement of suPAR and of its different forms during sepsis has, therefore, not been well defined. suPAR does not appear to be superior to other biomarkers, like CRP and PCT, in diagnosing sepsis. The independent predictive value of suPAR levels for outcome is more clearly established. suPAR levels may, therefore, be useful for triaging of patients for ICU admission, as high suPAR levels may indicate the need for more intense monitoring and treatment. The monitoring of suPAR levels during therapy needs further study to determine whether this biomarker could be of use in guiding therapeutic decisions. Finally, taking into account the present lack of a 'perfect' unique biomarker, further studies are warranted to evaluate the usefulness of combining several of the available biomarkers to improve their singular positive predictive values.

## List of abbreviations

APACHE II: Acute Physiology and Chronic Health Evaluation II; ART: antiretroviral treatment; AUROC: area under the receiver operating characteristic curve: CCHF: Crimean-Congo hemorrhagic fever; CNS: central nervous system; CRP: C- reactive protein; CSF: cerebrospinal fluid; PCT: procalcitonin; SAPS: Simplified Acute Physiology Score; SOFA: sequential organ failure assessment; suPAR: soluble urokinase-type plasminogen activator receptor.

## Competing interests

The authors declare that they have no competing interests.

## Authors' contributions

KD and CC researched the literature and drafted the manuscript. SS critically revised the content. JLV corrected the draft manuscript and critically revised the content. All authors approved the final version.

## Pre-publication history

The pre-publication history for this paper can be accessed here:

http://www.biomedcentral.com/1741-7015/10/2/prepub
